# Effects of childhood experience with nature on tolerance of urban residents toward hornets and wild boars in Japan

**DOI:** 10.1371/journal.pone.0175243

**Published:** 2017-04-07

**Authors:** Tetsuro Hosaka, Koun Sugimoto, Shinya Numata

**Affiliations:** Department of Tourism Science, Graduate School of Urban Environmental Sciences, Tokyo Metropolitan University, 1–1 Minami-Osawa, Hachioji, Tokyo, Japan; Waseda University, JAPAN

## Abstract

Urban biodiversity conservation often aims to promote the quality of life for urban residents by providing ecosystem services as well as habitats for diverse wildlife. However, biodiversity inevitably brings some disadvantages, including problems and nuisances caused by wildlife. Although some studies have reported that enhancement of nature interaction among urban children promotes their affective attitude toward of favorable animals, its effect on tolerance toward problem-causing wildlife is unknown. In this study, we assessed the tolerance of 1,030 urban residents in Japan toward hornets and wild boar, and analyzed the effects of childhood experience with nature on tolerance using a structural equation model. The model used sociodemographic factors and childhood nature experience as explanatory variables, affective attitude toward these animals as a mediator, and tolerance as a response variable. The public tolerance toward hornets and boars was low; over 60% of the respondents would request the removal of hornets and wild boar from nearby green spaces by government services, even when the animals had not caused any damage. Tolerance was lower in females and elderly respondents. Childhood experience with nature had a greater influence on tolerance than did sociodemographic factors in the scenario where animals have not caused any problems; however, its effect was only indirect via promoting positive affective attitude toward wildlife when the animals have caused problems. Our results suggest that increasing people’s direct experience with nature is important to raise public tolerance, but its effect is limited to cases where wildlife does not cause any problems. To obtain wider support for conservation in urban areas, conservationists, working together with municipal officials, educators and the media, should provide relevant information on the ecological functions performed by problem-causing wildlife and strategies for avoiding the problems that wildlife can cause.

## Introduction

As more than half the world’s population now lives in cities, urban biodiversity aims to promote the quality of life for urban residents through ecosystem services [1]. Ecosystem services in urban areas include air filtering, micro-climate regulation, noise reduction, rainwater drainage, sewage treatment, and recreational and cultural opportunities [2]. Interactions with wildlife can provide us with substantial advantages for our health and well-being [3]. Moreover, urban biodiversity conservation is also expected to prevent the cycle of “extinction of experience”; i.e., people lacking opportunities to interact with nature are less likely to value and appreciate it, which leads to a decline in public support for conservation activities and further degradation of the natural environment [4, 5].

However, the outcomes of more natural ecosystems and richer biodiversity are not always positive for human well-being and can even be negative or harmful, and these are known as ecosystem disservices [6, 7]. The negative aspects include the emission of allergens, damage to infrastructure and people by falling branches, fear of dark and wooded areas at night, and an increase in nuisance wildlife harming humans [8, 9]. Human–wildlife conflict occurs not only in rural or agricultural landscapes but also in urban settings, and often in the vicinity of green spaces [10]. Our recent study showed a positive correlation between the size of urban green spaces and abundance of [11] and conflicts with hornets [12].

In most cases, the actual problems caused by urban wildlife are minor, and severe physical damage, such as injury or fatality due to an attack, are rare [10]. However, even minor nuisances can be highly relevant in the urban setting of an affluent industrialized society [13], and even rare wildlife-related problems, if serious, can dramatically alter the public perception of wildlife and its habitat [14]. In Tokyo, a recent resurgence of dengue fever after 70 years led, in 2014, to an extermination program of mosquitoes by intensive insecticide spraying in some large parks where biodiversity conservation programs had been established [15]. In the USA, urban wildlife issues cause an estimated $8.6 billion worth of damage, but the systematic and proactive control of wildlife is generally impractical because this would cost much more [10]. If requests for extermination of wildlife and compensation for wildlife-associated problems increase, the financial burden for governments could be serious.

Thus, understanding public attitudes and tolerance toward problem-causing wildlife is important to obtain the support of the public for conservation programs. A large number of studies have measured perceptions, attitudes, and tolerance of urban residents toward wildlife (often large or carnivorous mammals). Factors including sex, age, ethnicity, wealth, and education can all affect attitudes toward wildlife [14]. Some empirical studies have also demonstrated that experience with nature can positively influence children’s affective attitudes (a psychological tendency to respond positively or negatively toward certain object; e.g., likes and dislikes) toward wildlife including unpopular organisms [16–18], although whether the effect of childhood nature experience lasts until adulthood remains unclear. Furthermore, one’s affective attitude toward wildlife may positively affect tolerance toward problem-causing wildlife. For example, problems caused by popular wildlife were more acceptable to local residents than were those caused by unpopular wildlife [19]. Therefore, increasing interactions with nature through urban biodiversity conservation may not only enhance public affective attitudes toward wildlife but may also increase public tolerance of problem-causing wildlife. However, the effects of childhood experience on affective attitudes and tolerance toward problem-causing wildlife and the strength of the effects, compared to the effects of sociodemographic factors, have never been examined.

**Fig 1:**
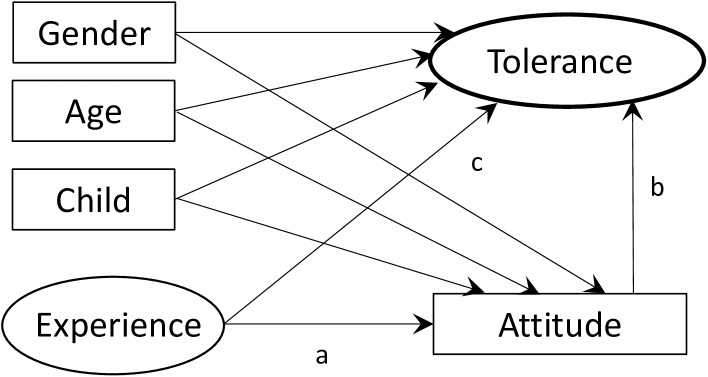
Model of the hypothesized paths of the relationships among sociodemographic factors (sex, age, and having children or no children), childhood experience with nature (Experience), affective attitude toward hornets and wild boar (Attitude), and tolerance to having this wildlife nearby (Tolerance). The total effect is the sum of an indirect effect (e.g., a × b) and a direct effect (e.g., c).

We constructed a structural equation model (SEM; Fig 1) based on the results of the empirical studies mentioned above. Here, we hypothesized that childhood experience with nature (Experience) positively affects affective attitudes (Attitude) toward problem-causing wildlife based on [16, 17] (Path a) and that affective attitude positively affects people’s tolerance toward the problems caused by wildlife (Tolerance) based on [19, 20] (Path b). Experience may also have a direct effect on Tolerance (Path c). We included some sociodemographic parameters (i.e., sex, age, having children or no children, and income) to control for their potential confounding effects on affective attitude and tolerance, and to evaluate the importance of the experience relative that of sociodemographic factors. Our study addressed the following question: Does Experience affect Tolerance directly or indirectly via Attitude, and if so, how strong are the effects compared to the effects of sociodemographic parameters?

## Methods

### Study site

We conducted a questionnaire survey in the capital area of Japan, Tokyo and its surrounding areas including Chiba, Saitama, and Kanagawa prefectures. The area is home to the world’s largest urban population (38 million inhabitants) [21].

Biodiversity conservation is a new keyword in the design and management of urban green spaces in Tokyo. Although the total area of green spaces in the city has continually decreased since the 1960s through the conversion of forested and agricultural land, the rate of decline has decreased recent years [12]. Following the adoption of the Nagoya Protocol of the Convention on Biological Diversity in 2010, the Tokyo Metropolitan Government and individual local governments established strategies for urban biodiversity conservation with plans to increase the size and value of green spaces for local biodiversity through improvements in their quality and connectivity [22]. For example, the Tokyo Metropolitan Government planned to create 1,000 ha of new green spaces over a period of 10 years [22].

### Questionnaire

We hired an Internet research company (Macromill, Inc., Tokyo) to conduct a web-based questionnaire survey on Experience, Attitude, and Tolerance among residents in the study area in January 2016. Macromill strictly follow the privacy policy and research guidelines of the Essential Organisation for encouraging, advancing and elevating Market Research worldwide (ESOMAR) and the Japan Marketing Research Association, and holds the “PrivacyMark” certification of the Japan Information Processing and Development Center (JIPDEC) [23]. The data are rendered completely anonymous when we obtain the data, and the process holds no potential risks to individuals or individual privacy. The participants provided their informed consent online to participate in this study prior to the present survey. Therefore, we did not seek approval by an institutional review board (ethics committee) for the study.

The questionnaire was answered by 1,030 urban and suburban residents (20‒69 years old) in the study area. We collected equal numbers of responses for each sex and age group (e.g., 103 males of 20‒29 years old, 103 females of 20‒29 years old, ···, 103 females of 60‒69 years old). The respondents were similar to those participating in other nation-wide surveys such as public opinion polls in most characteristics, including frequency of involvement in outdoor activities and environmental concerns,but differed in frequency of Internet use [24].

To quantify Experience, respondents answered a self-report item about the frequency with which they used green spaces (including four types of area: parks, forests, farmland, and rivers/ocean), and were involved in nature-related activities (insect catching, fishing, collecting wild flowers and fruits, tree climbing, and swimming in rivers/ocean) during their childhood (aged ≤ 12 years)(S1 Table). These activities have been common among Japanese children during the past 70 years or longer [25]. Respondents replied based on a 5-point scale ranging from 1 (never) to 5 (very often). Although the retrospective self-report approach limited in quantifying the actual frequency of activities, we adopted this method following previous studies [26, 27] due to the ease of answering for respondents and the difficulty of collecting reliable objective data.

To quantify Attitude, respondents were asked to rate the level of affective attitude (i.e., likes and dislikes) toward two taxonomically distant animals, hornets (*Vespa* spp.) and wild boar (*Sus scrofa*), using a 5-point scale ranging from 1 (dislike) to 5 (like), with 3 as a neutral point. Hornets and wild boar are common nuisance animals in urban and suburban areas of Japan [28, 29]. Approximately 27,000 pest consultations (17% of the total) to local governments were related to hornet wasps in Tokyo during 2010‒2014 [12]. Annually, 13–45 people in the country were killed by hornet stings during 1989–2013, exceeding the number of deaths attributed to other animals [30]. Wild boar are responsible for the second largest amount of damage to agricultural crops in rural areas after deer [31]. Moreover, recently, boar have begun to appear in urban landscapes near forest edges [32] and cause problems in gardens and garbage stations, as well as injuries to people [33]. We also asked whether the respondents were familiar with the animals, regardless of whether they had encountered them directly. When they were not familiar with the animal, they were not asked to respond to the question about their Attitude toward that animal.

Tolerance toward problem-causing wildlife was assessed by measuring the level of acceptability associated with wildlife management actions that entailed various levels of control over the animals in six different scenarios (H1‒H3 for hornet, and B1‒B3 for wild boar) related to wildlife problems (Table 1). The scenarios reflected actual problem situations with respect to each animal in Japan. The problems caused by the animals increased in severity from H1 (presence only) to H3 (severe injury) for hornets, and from B1 (presence only) to B3 (severe injury) for wild boar. For each scenario, five possible management strategies were listed: (m1) do nothing, (m2) monitor the situation, (m3) alert the public, (m4) translocate the animal or nest, and (m5) trap and eliminate the animal. Respondents chose a level of tolerance for each management strategy based on a 5-point scale, ranging from 1 (totally unacceptable) to 5 (totally acceptable) with 3 as a neutral point. These measures are similar to acceptability measures used in previous studies [34, 35].

**Table 1:** Scenarios of human–wildlife interactions (hornets: H1‒H3, wild boar: B1‒B3) with different levels of severity to measure public tolerance toward the wildlife.

No.	Scenario
H1	Hornets have flown to a park near your house. There is a chance that park users will encounter them.
H2	Hornets have made a nest in a park near your house. There is a chance that park users can be stung by them.
H3	Hornets nesting in a park near your house have attacked and severely injured a park user.
B1	Wild boar live in a green space near your house. There is a chance that residents will encounter them.
B2	Wild boar living in a green space near your house have disturbed gardens, farms, and garbage stations.
B3	Wild boar living in a green space near your house have attacked and severely injured a resident.

### Analysis

We used the mean scores of all items for Experience as the Experience score because the Cronbach’s α (a measure of internal consistency among a group of questionnaire items) was 0.87, which is well above the generally acceptable level (α = 0.65) for these items to be combined into an additive index [36]. As a measure of Tolerance, we used the mean scores of m1‒m3 for each scenario as the Tolerance score because the scores represented how acceptable the respondents were toward the animals without removal. The Cronbach’s α for m1‒m3 was sufficiently high for all of the scenarios: 0.86 (H1), 0.89 (H2), 0.90 (H3), 0.87 (B1), 0.89 (B2), and 0.91 (B3).

To investigate our main research question, we used a mediation analysis in the SEMs using the *lavaan* package [37] for R version 3. 3. 1. We constructed a SEM using sociodemographic factors and Experience as explanatory variables, Attitude as a mediator, and Tolerance as a response variable (Fig 1). For the sociodemographic parameters, we used sex, age, and having children or no children, which are known to affect public attitudes toward wildlife [38, 39]. Education and media coverage would also strongly influence public attitudes toward wildlife [38, 40]; however, their effects would be highly dependent on their contents, contexts and the tools used [41, 42]. Since people are exposed on a daily basis to a variety of information from the mass media and environmental education programs, we believe that it would be difficult to quantify their effects in our study; thus, we did not include parameters related to the education and media exposure of the respondents. These sociodemographic parameters have previously been used to assess public attitudes toward problem-causing wildlife [22]. Although we included private and household income in the preliminary analysis, these variables had no significant effects on Attitude and Tolerance in any of the scenarios; thus, they were excluded from the analyses. We constructed a full model in which Experience and the sociodemographic parameters both had direct effects on Tolerance and indirect effects via Attitude (Fig 1). We calculated standardized coefficients for every path to compare the strength of the correlations. An indirect effect is the product of the coefficient for a path from an explanatory variable to a mediation variable, and that from a mediation variable to a response variable (e.g., a × b in Fig 1). A total effect is the sum of a direct effect and the indirect effect (e.g., c + a × b in Fig 1). The standard errors for the indirect effects were estimated using both the Delta and bootstrap methods [37], but we only report the results based on the Delta method because both methods yielded similar results. We deleted any nonsignificant paths from the full model using a stepwise process to obtain the best model. The model fitness was assessed as good based on multiple criteria: the χ^2^ goodness-of-fit statistic and the P-value (> 0.05 for a good fit), the comparative fit index (CFI: 0.95‒1.00 for a good fit), root mean square error of approximation (RMSEA: 0.000‒0.050 for a good fit), and standardised root mean square residual (SRMR: 0.000–0.080 for a good fit) (S2 Table). A data set underlying our analyses are available at S3 Table.

## Results

**Fig 2:**
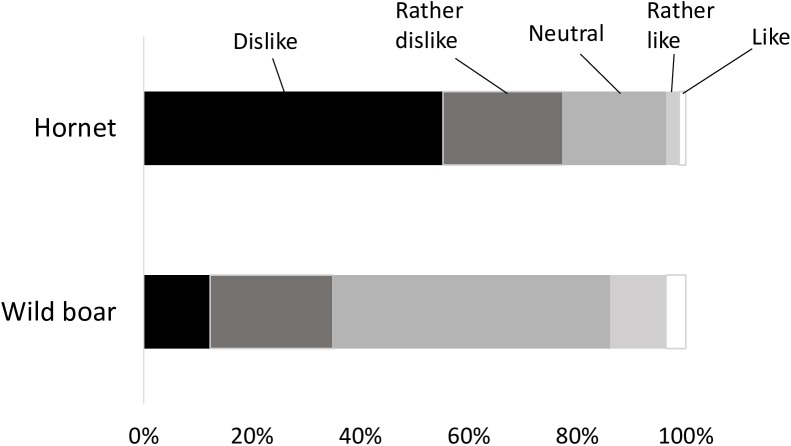
Proportions of respondents who liked/disliked hornets and wild boar.

Most respondents (77%) either disliked or ‘rather disliked’ hornets (Fig 2). On the other hand, more than half of the respondents (51%) showed a neutral Attitude toward wild boar, although only 14% of respondents either liked or ‘rather liked’ boar.

**Fig 3:**
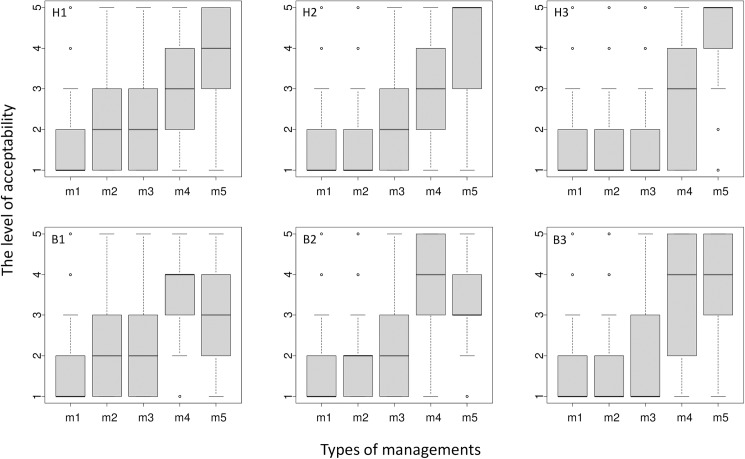
Box-and-whisker plots for scores indicating acceptability of five wildlife management strategies (m1: do nothing; m2: monitor the situation; m3: alert the public; m4: translocate the animals or nest; m5: trap and eliminate the animals) for six scenarios (hornets: H1‒H3, wild boar: B1‒B3). Box-and-whisker plots indicate the first, second (the median) and third quartile by the bottom, the bold band, and top of the box, respectively. The upper and lower ends of whiskers indicate the minimum and maximum within the 1.5 interquartile range, respectively. Points indicate outliers beyond the 1.5 interquartile range. See Table 1 for details of the scenarios.

The level of tolerance toward problem-causing wildlife was low in scenarios both with and without resulting damage. Public acceptance of the management of damage-causing wildlife was lowest for the least severe management type (do nothing) in all of the scenarios (only 2.3‒6.2% of respondents felt that that was totally acceptable or acceptable; Fig 3). Even when the wildlife caused no problems (scenarios H1 and B1), 76% and 77% of the respondents deemed it unacceptable for the local government to do nothing about the presence of hornets and wild boar, respectively. The most severe management solution (elimination) was most supported in all of the hornet scenarios (H1: 66%, H2: 74%, H3: 76%). The second most severe management solution (translocation) was the most supported in both boar scenarios (B1: 60%, B2: 60%), although elimination of boar was most supported when they attacked and injured people (B3: 60%).

**Fig 4:**
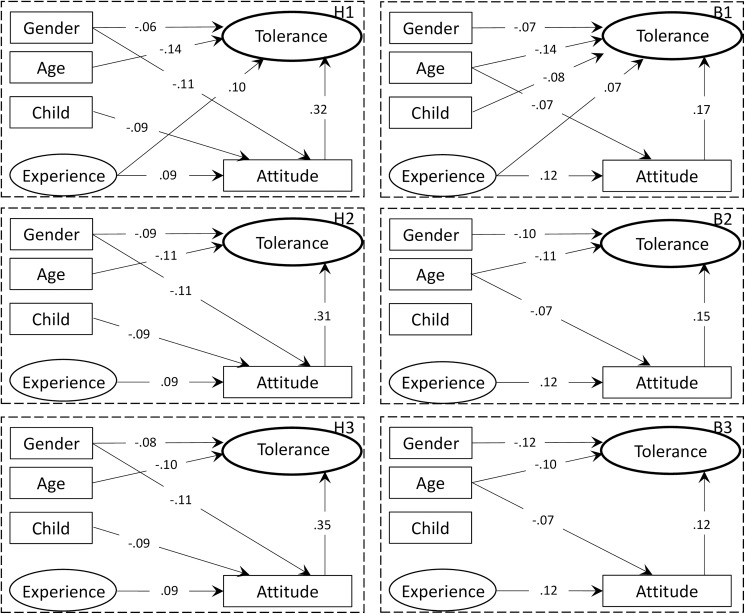
The best SEM using sociodemographic factors; i.e., sex (male = 0, female = 1), age, having children (= 1) or no children (= 0), and the level of childhood experience with nature (Experience), affective attitude (Attitude), and tolerance toward hornets (H1‒H3) and wild boar (B1‒B3) after deleting the nonsignificant paths from the full model (Fig 1). See Table 1 for details of the scenarios.

Experience positively correlated with Attitude toward both hornets and wild boar (Fig 4). Among the socio-demographic factors, sex (female) and having children negatively correlated with Attitude towards hornets, and age negatively correlated with Attitude toward wild boar. Of all of the paths concerning Attitude toward wildlife, the absolute value of the standardized path coefficients of Experience was the second highest for the hornet scenarios and highest for the boar scenarios. This suggests that the effect of Experience on Attitude is similar to or greater than those of the socio-demographic factors.

Attitude positively correlated with Tolerance in all of the scenarios for hornets and wild boar (Fig 4). Among the socio-demographic factors, sex (female) and age negatively correlated with Attitude in all of the scenarios. Having children had a significant negative correlation with Tolerance only in scenario B1. Of all of the paths concerning Tolerance, the absolute value of the standardized path coefficients of Attitude was greater than those of the other variables for all hornet and boar scenarios, except for B3.

A direct path from Experience to Tolerance was only significant for H1 and B1 (Fig 3, Table 2), but an indirect path from Experience to Tolerance via Attitude was significant for all of the scenarios (Table 2). Although the total effect of Experience on Tolerance was greatest or second greatest among the explanatory variables in scenarios H1 and B1, it was not significant in any other scenarios. The direct effects of sex and age on Tolerance were stronger than their indirect effects (Table 2). Similarly, sex and age had a greater total effect on Tolerance than that of Experience in all scenarios, except H1 and B1. The total effects of sex became more negative as the severity of the problem increased, while that of age became less negative.

**Table 2:** The standardized path coefficients for direct, indirect, and total effects on tolerance toward hornets (H1‒H3) and wild boar (B1‒B3) in each scenario. Male and no children are the reference categories for sex and having children, respectively.

Scenario	Variable	Direct		Indirect		Total	
H1	Sex	-0.063	*	-0.037	**	-0.100	***
	Age	-0.136	***	0.013		-0.123	***
	Children	-0.005		-0.030	*	-0.035	
	Experience	0.095	**	0.028	**	0.123	***
H2	Sex	-0.087	**	-0.036	**	-0.123	***
	Age	-0.105	**	0.013		-0.093	**
	Children	-0.013		-0.029	*	-0.042	
	Experience	0.024		0.027	**	0.051	
H3	Sex	-0.084	**	-0.040	**	-0.124	***
	Age	-0.096	**	0.014		-0.082	*
	Children	-0.015		-0.032	**	-0.047	
	Experience	0.013		0.030	**	0.043	
B1	Sex	-0.068	*	-0.004	*	-0.073	*
	Age	-0.142	***	-0.012		-0.154	***
	Children	-0.084	*	-0.004		-0.088	*
	Experience	0.071	*	0.021	**	0.092	**
B2	Sex	-0.103	**	-0.004		-0.107	**
	Age	-0.113	**	-0.010		-0.124	***
	Children	-0.060		-0.003		-0.063	
	Experience	0.019		0.018	**	0.037	
B3	Sex	-0.116	***	-0.003		-0.119	***
	Age	-0.100	**	-0.008		-0.108	**
	Children	-0.053		-0.003		-0.056	
	Experience	0.028		0.014	**	0.042	

Significance levels are

*p < 0.05

**p < 0.01

***p < 0.001.

## Discussion

### Affective attitude and tolerance toward problem-causing wildlife

As expected, many urban residents did not like hornets and wild boar, although more people disliked hornets than boar. This might be due to taxonomic differences because insects are globally less popular than mammals [10, 43].

The public attitude to having these animals in their vicinity was similarly negative for hornets and wild boar. More than three quarters of the respondents felt it unacceptable for the government to do nothing regarding the presence of hornets and wild boar in the parks and green spaces, and wanted the elimination of these animals. This may reflect a general perception that urban wildlife is a nuisance [10]. Other studies have also suggested that urban residents are less tolerant toward wild animals than rural residents are, or that they switch to negative from positive when the animals cause problems [44, 45, 46]. Because the wildlife tolerance levels were measured for wildlife inhabiting nearby green spaces without any government intervention, the low tolerance levels indicate the high public dependency on government services to deal with wildlife problems. To avoid excessive costs and manpower, it is critical for governments to develop strategies to combat this low tolerance among urban residents toward problem-causing wildlife, whose numbers are likely to increase with the increasing green urban spaces [11].

### Socio-demographic factors affecting acceptance of nuisance wildlife

Sex and age had significant effects on Attitude and Tolerance to problem-causing wildlife; females and older people had more negative attitudes and lower Tolerance than males and younger people. Sex was the most influential explanatory variable on Tolerance in three of the six scenarios, particularly when the scenarios were more severe (e.g., resulting in severe injury). Age was also an influential explanatory variable on Tolerance in three scenarios. Previous studies have also reported more negative attitudes toward nuisance mammals and insects in females than in males [43, 44, 47] and in older people than in younger ones [28, 40, 44]. Concern about environmental risks is higher in females than in males [48]. The low tolerance among the elderly people may be due to their utilitarian and dominionistic views toward animals [39], or a lack of manpower and knowledge to prevent problems associated with wildlife [28].

### Effect of experience on attitude and tolerance

The SEM analysis revealed that childhood experience with the natural environment positively affected Attitude and that Attitude had a positive effect on Tolerance toward hornets and boar in all of the scenarios (Fig 4). Experience was most influential or second most influential on Tolerance when the animals did not cause any problems (H1 and B1). This highlights the importance of increasing opportunities for urban children to interact with nature. Previous studies have also reported a positive relationship between experience with nature and an interest in environmentally friendly practices [49], positive perceptions of the natural environment and outdoor recreational activities [50], and positive affective attitude toward wild animals [16, 17] in children. Our study demonstrated that childhood experience with nature remains influential on their Attitude and Tolerance to problem-causing wildlife later in life. Therefore, conservation programs in urban areas should place greater emphasis on encouraging engagement of children with nature to promote positive affective attitudes and tolerance toward wildlife.

Childhood experience, however, had no significant direct or total effect on Tolerance when the wildlife caused problems, although it had a significant indirect effect via Attitude. This suggests that the effects of childhood experience on Tolerance are limited to cases where wildlife does not cause any problems, while the effects of Attitude remain important. Therefore, enhancing affective attitudes toward problem-causing wildlife offers an option for increasing public tolerance. Because knowledge about wildlife can often be at the root of liking or disliking animals [51], education about the ecological functions and cultural importance of wildlife, including animals that are seen as pests, is important. For example, public information campaigns and educational programs successfully changed public attitudes toward bobcats [52], crocodiles [41], snakes [18], and tarantulas [53]. Additionally, people should be educated about how to avoid problems caused by wildlife with appropriate information about risk, as perceived risk is often much higher than the actual risk [14, 52]. This may be particularly important for females and elderly people, who showed lower tolerance in our study. Hornets usually do not attack people unless people attack or frighten them first, and farmers traditionally allowed them to make nests under the eaves of houses, as they are important predators of agricultural pests [54]. Such knowledge may reduce the fear and increase public tolerance toward hornets in urban areas. In contrast, many TV and radio broadcasts about hornets highlight only their dangerous aspects [29]. This type of broadcast is likely to reduce tolerance toward hornets, and has led to recent increases in the number of consultations about, and exterminations of, hornets in many cities in Japan [29]. Greater knowledge among the urban population about ecosystem services that acknowledge the problems caused by wildlife, e.g., the ecological importance of problem-causing wildlife, actual risks, and preventive measures to avoid or mitigate problems, can contribute to increased tolerance [55].

We also wish to mention a few caveats. First, our retrospective self-report approach is somewhat subjective and is therefore limited in quantifying the actual frequency of childhood activities. Prospective and longitudinal approaches that record the actual frequency of children’s nature activities and track these individuals over at least 20 years would be more useful [26], although this would not be easy. Therefore, more accurate interpretation of the results in this study would be that people who think themselves frequently engaged in nature activities during childhood showed higher affective attitudes and tolerance than those who do not think so. However, we believe that this still has important implications for understanding the relationships between childhood nature experience and attitudes toward problem-causing wildlife. Second, it might not be valid to generalize our study, which focuses on only two animals (hornets and wild boar), to all problem-causing wildlife. Although these animals represent major nuisance animals in urban areas in Japan, the main animals causing problems in other countries can be largely different. Tolerance, however, is often dependent on the impacts rather than on the animal species [52]. Therefore, we believe that our approach using multiple scenarios of different impacts also provides useful information about tolerance toward problem-causing wildlife and the factors that influence it in urban areas in countries other than Japan. Finally, many of the path coefficients were not large, although they were significant. This implies that large variances in affective attitude and tolerance remain unexplained. As discussed above, education and media coverage may have significant effects on public attitudes toward wildlife [39, 40]. Further study is needed to identify which types of education and media coverage that are most effective in changing public tolerance toward problem-causing wildlife (e.g., [41]).

## Conclusion

Biodiversity conservation in urban areas is increasingly highlighted to promote habitat quality for both humans and wildlife as urbanization accelerates around the world. However, increasing biodiversity in urban areas will inevitably bring various disadvantages, including nuisances and problems caused by wildlife. Our study showed that urban residents generally had low tolerance toward problem-causing wildlife and high dependency on governments to solve the problems. This should be kept in mind when governments launch local or regional strategies for biodiversity conservation, as those strategies often only consider the positive side of biodiversity and overlook negative public attitudes toward urban wildlife. The growing lack of childhood experience with nature may further reduce the tolerance in the future, which may present an obstacle to urban biodiversity conservation. To obtain wider support for conservation in urban areas, biologists working closely with municipal officials, educators and media, should provide relevant information on the ecological functions of problem-causing wildlife and strategies to avoid these problems.

## Supporting information

S1 Table Questionnaire items used in the present study (the original version is in Japanese). (DOCX)

S2 Table The fitness measures of the SEMs for each scenario. (DOCX)

S3 Table A data set for the present study. (CSV)
