# Supplementary material for: Effects of childhood experience with nature on tolerance of urban residents toward hornets and wild boars in Japan
Source: PLoS One. 2017 Apr 7;12(4):e0175243. doi: 10.1371/journal.pone.0175243 (PMC5384670; doi:10.1371/journal.pone.0175243)
Supplement: S1 Table — (DOCX) [file pone.0175243.s001.docx]

S1 Table

Please answer your childhood (12 years old or younger) experience with nature

Q1 How often did you play in the following natural environments? Please choose one of the 1-5.

|  | Never | Seldom | Sometimes | Often | Very often |
| --- | --- | --- | --- | --- | --- |
| 1. Parks | 1 | 2 | 3 | 4 | 5 |
| 2. Forest | 1 | 2 | 3 | 4 | 5 |
| 3. Farmlands | 1 | 2 | 3 | 4 | 5 |
| 4. Rivers/oceans | 1 | 2 | 3 | 4 | 5 |

Q2 How often were you involved in the following nature-related activities? Please choose one of the 1-5.

|  | Never | Seldom | Sometimes | Often | Very often |
| --- | --- | --- | --- | --- | --- |
| 1. Insect catching | 1 | 2 | 3 | 4 | 5 |
| 2. Fishing | 1 | 2 | 3 | 4 | 5 |
| 3. Collecting wild flowers and fruits | 1 | 2 | 3 | 4 | 5 |
| 4. Tree climbing | 1 | 2 | 3 | 4 | 5 |
| 5. Swimming in the rivers/oceans | 1 | 2 | 3 | 4 | 5 |

Q3 To what extent do you know about the following animals? Please choose one of the 1-5.

|  | Do not know | Only know by name | Have watched picture or movie | Have watched in the zoo or insectarium | Have watched in the wild |
| --- | --- | --- | --- | --- | --- |
| Hornet | 1 | 2 | 3 | 4 | 5 |
| Boar | 1 | 2 | 3 | 4 | 5 |

Q4 To what extent do you like or dislike the following animals? Please choose one of the 1-5.

|  | Dislike | Rather dislike | Neutral | Rather like | Like |
| --- | --- | --- | --- | --- | --- |
| Hornet | 1 | 2 | 3 | 4 | 5 |
| Boar | 1 | 2 | 3 | 4 | 5 |

Q5. This part focuses on questions about wildlife-human situations that can happen in Singapore now or in the future. Below you will find three different situations involving hornets, macaques and pythons. For every situation there are five different possible management actions. Please tick the relevant box to rate your acceptance on each management action.

Hornet

H1 Hornets have flown to a park near your house. There is a chance that park users will encounter them.

| How unacceptable or acceptable is it if wildlife agencies | Totally Unacceptable | Slightly Unacceptable | Neutral | Slightly acceptable | Totally acceptable |
| --- | --- | --- | --- | --- | --- |
| m1. Do nothing | 1 | 2 | 3 | 4 | 5 |
| m2. Only monitor the situation | 1 | 2 | 3 | 4 | 5 |
| m3. Only educate the public | 1 | 2 | 3 | 4 | 5 |
| m4. Translocate the hornet | 1 | 2 | 3 | 4 | 5 |
| m5. Trap and eliminate the hornet | 1 | 2 | 3 | 4 | 5 |

H2 Hornets have made a nest in a park near your house. There is a chance that park users can be stung by them.

| How unacceptable or acceptable is it if wildlife agencies | Totally Unacceptable | Slightly Unacceptable | Neutral | Slightly acceptable | Totally acceptable |
| --- | --- | --- | --- | --- | --- |
| m1. Do nothing | 1 | 2 | 3 | 4 | 5 |
| m2. Only monitor the situation | 1 | 2 | 3 | 4 | 5 |
| m3. Only educate the public | 1 | 2 | 3 | 4 | 5 |
| m4. Translocate the wasp | 1 | 2 | 3 | 4 | 5 |
| m5. Trap and eliminate the wasp | 1 | 2 | 3 | 4 | 5 |

H3 Hornets nesting in a park near your house have attacked and severely injured a park user.

| How unacceptable or acceptable is it if wildlife agencies | Totally Unacceptable | Slightly Unacceptable | Neutral | Slightly acceptable | Totally acceptable |
| --- | --- | --- | --- | --- | --- |
| m1. Do nothing | 1 | 2 | 3 | 4 | 5 |
| m2. Only monitor the situation | 1 | 2 | 3 | 4 | 5 |
| m3. Only educate the public | 1 | 2 | 3 | 4 | 5 |
| m4. Translocate the wasp | 1 | 2 | 3 | 4 | 5 |
| m5. Trap and eliminate the wasp | 1 | 2 | 3 | 4 | 5 |

Wild boar

B1 Wild boar live in a green space near your house. There is a chance that residents will encounter them.

| How unacceptable or acceptable is it if wildlife agencies | Totally Unacceptable | Slightly Unacceptable | Neutral | Slightly acceptable | Totally acceptable |
| --- | --- | --- | --- | --- | --- |
| m1. Do nothing | 1 | 2 | 3 | 4 | 5 |
| m2. Only monitor the situation | 1 | 2 | 3 | 4 | 5 |
| m3. Only educate the public | 1 | 2 | 3 | 4 | 5 |
| m4. Translocate the wasp | 1 | 2 | 3 | 4 | 5 |
| m5. Trap and eliminate the wasp | 1 | 2 | 3 | 4 | 5 |

B2 Wild boar living in a green space near your house have disturbed gardens, farms, and garbage stations.

| How unacceptable or acceptable is it if wildlife agencies | Totally Unacceptable | Slightly Unacceptable | Neutral | Slightly acceptable | Totally acceptable |
| --- | --- | --- | --- | --- | --- |
| m1. Do nothing | 1 | 2 | 3 | 4 | 5 |
| m2. Only monitor the situation | 1 | 2 | 3 | 4 | 5 |
| m3. Only educate the public | 1 | 2 | 3 | 4 | 5 |
| m4. Translocate the wasp | 1 | 2 | 3 | 4 | 5 |
| m5. Trap and eliminate the wasp | 1 | 2 | 3 | 4 | 5 |

B3 Wild boar living in a green space near your house have attacked and severely injured a resident.

| How unacceptable or acceptable is it if wildlife agencies | Totally Unacceptable | Slightly Unacceptable | Neutral | Slightly acceptable | Totally acceptable |
| --- | --- | --- | --- | --- | --- |
| m1. Do nothing | 1 | 2 | 3 | 4 | 5 |
| m2. Only monitor the situation | 1 | 2 | 3 | 4 | 5 |
| m3. Only educate the public | 1 | 2 | 3 | 4 | 5 |
| m4. Translocate the wasp | 1 | 2 | 3 | 4 | 5 |
| m5. Trap and eliminate the wasp | 1 | 2 | 3 | 4 | 5 |

Please answer about yourself

Q6 Gender

1. Male

2. Female

Q7 Age

( ) years old

Q8 Do you have children?

1. Yes

2. No

Q9 Annual household income

1. Less than 2 million yen

2. 2~4 million yen

3. 4~6 million yen

4. 6~8 million yen

5. 8~10 million yen

6. 10~12 million yen

7. 12~15 million yen

8. 15~20 million yen

9. 20 million yen or more

Q10 Annual personal income

1. Less than 2 million yen

2. 2~4 million yen

3. 4~6 million yen

4. 6~8 million yen

5. 8~10 million yen

6. 10~12 million yen

7. 12~15 million yen

8. 15~20 million yen

9. 20 million yen or more
