# Supplementary material for: Effects of childhood experience with nature on tolerance of urban residents toward hornets and wild boars in Japan
Source: PLoS One. 2017 Apr 7;12(4):e0175243. doi: 10.1371/journal.pone.0175243 (PMC5384670; doi:10.1371/journal.pone.0175243)
Supplement: S2 Table — (DOCX) [file pone.0175243.s002.docx]

S2 Table.

| Scenario | *χ2* | *df* | *p* | CFI | RMSEA | SRMR |
| --- | --- | --- | --- | --- | --- | --- |
| H1 | 1.4 | 2 | 0.50 | 1.000 | 0.000 | 0.007 |
| H2 | 2.1 | 3 | 0.56 | 1.000 | 0.000 | 0.009 |
| H3 | 1.7 | 3 | 0.63 | 1.000 | 0.000 | 0.008 |
| B1 | 1.2 | 2 | 0.55 | 1.000 | 0.000 | 0.008 |
| B2 | 1.0 | 2 | 0.61 | 1.000 | 0.000 | 0.008 |
| B3 | 1.4 | 2 | 0.51 | 1.000 | 0.000 | 0.009 |
